# A Narrative Review of Bioactive Glass-Loaded Dental Resin Composites

**DOI:** 10.3390/jfb13040208

**Published:** 2022-10-28

**Authors:** Jiaojiao Yun, Michael Francis Burrow, Jukka Pekka Matinlinna, Yan Wang, James Kit Hon Tsoi

**Affiliations:** 1 Dental Materials Science, Division of Applied Oral Sciences and Community Dental Care, Faculty of Dentistry, The University of Hong Kong, Hong Kong SAR, China; 2 Prosthodontics, Division of Restorative Dental Sciences, Faculty of Dentistry, The University of Hong Kong, Hong Kong SAR, China; 3 Division of Dentistry, School of Medical Sciences, University of Manchester, Manchester M13 9PL, UK; 4 Department of Prosthodontics, Guanghua School of Stomatology, Hospital of Stomatology, Guangdong Key Laboratory of Stomatology, Sun Yat-sen University, Guangzhou 510055, China

**Keywords:** bioactive glass, resin composites, physicochemical properties, mechanical properties, mineralization ability, biological responses

## Abstract

This review aims to provide a comprehensive analysis of the characterizations of bioactive glass (BAG)-loaded dental resin-based composite materials. Online databases (Web of Science, PubMed, and Science Direct) were used to collect data published from January 2011 to January 2022. Only BAG-containing resin adhesive and resin restorative composites are discussed in this narrative review. BAG-loaded resin composites exhibit excellent mineralization ability reflecting enhanced ion release, pH elevation, and apatite formation, especially regarding high BAG loading. This aids the anti-demineralization and remineralization of teeth. Furthermore, BAG-loaded resin composites demonstrated *in vitro* biocompatibility and antibacterial performance. It has been suggested that BAG fillers with small particle sizes and no more than 20 wt% in terms of loading amount should be used to guarantee the appropriate mechanical properties of resin composites. However, most of these studies focused on one or some aspects using different resin systems, BAG types, and BAG amounts. As such, this makes the comparison difficult, and it is essential to find an optimal balance between different properties. BAG-loaded resin composites can be regarded as bioactive materials, which present major benefits in dentistry, especially their capability in the bacterial inhibition, cell biocompatibility, anti-demineralization, and remineralization of teeth.

## 1. Introduction

The ultimate performance of the restored tooth structure highly depends on the use of durable, biocompatible, and aesthetically acceptable materials. In the current dental practice, contemporary resin-based composites (RBCs) (so-called resin composites) have been widely advocated and accepted as a replacement for dental amalgam restorations due to the absence of mercury release, superior aesthetics, minimal preparation procedure, the direct-filling technique, being thermally nonconductive, and the notable improvement in its mechanical properties [1].

**Figure 1:**
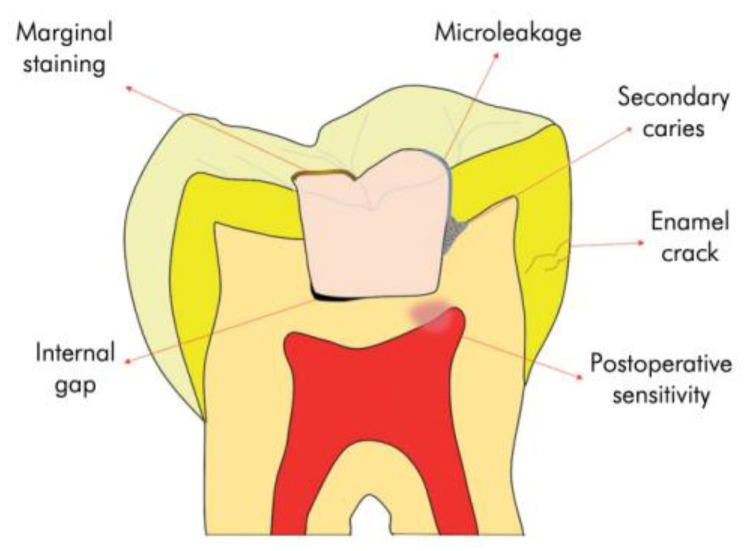
The schematic of the problems of polymerization shrinkage of resin composites. From [5].

Dental resin composites are a class of dental restorative materials that mainly consist of a highly cross-linked polymeric resin matrix reinforced by inorganic filler particles that are coated with silane coupling agents [2]. However, there is a major concern over current RBCs, namely the formation of a gap existing between the restorative material and tooth tissue caused by polymerization shrinkage (1.7–6 vol%) [3] (Figure 1). This gap can provide a potential pathway for bacteria, fluids, and ions to penetrate into the gap via microleakage, which may cause recurrent caries, and consequently fail the composite restoration [4,5]. Moreover, it has been reported that resin composites are more prone to accumulate biofilms and plaque on their surface compared with the tooth surface and other restorative materials [6,7]. As a result, biofilm accumulation is usually observed on the surface of restorative materials and at the interface between restorations and the cavity wall, which can then lead to recurrent caries. Furthermore, the accumulation of bacteria may consume saccharides and produce lactic acid as a byproduct which, in turn, could cause the demineralization of tooth tissues [8], as well as the degradation of the restorative materials, thus reducing the longevity of composite materials. Therefore, there is a need to develop functional restorative materials exhibiting functions, e.g., the formation of hydroxyapatite (HA) that can seal the marginal gap, repair and/or regenerate the defective dental tissue via remineralization, and incorporation of antibacterial agents into the resin composites that can reduce bacterial accumulation and biofilm formation.

Bioactive glass (BAG) has been shown to have a similar composition to the structure of teeth and natural bone and the ability to enhance bone formation [9,10], inhibit bacteria [11,12,13], and remineralize adjacent mineralized tissues [14,15], all of which are related to the dissolution of BAG. Once in contact with body fluids, the Na^+^ and Ca^2+^ from the BAG surface are exchanged with H^+^ from surrounding solutions yielding a negatively charged silica gel surface layer that leads to the formation of HA and, consequently, the formation of new bone [9,10]. Moreover, the release of OH^–^ results in an elevated local pH value adjacent to the material that is not well tolerated by many oral bacteria. At the same time, releasing ions (Ca^2+^, Na^+^, PO_4_^3−^, Si^4+^, etc.) can cause a high osmotic pressure leading to the destruction of the bacterial outer membrane and leakage of cell contents [16,17]. These remineralizing ions have also demonstrated a stronger ability to remineralize teeth and prevent the demineralization of teeth [14,15]. Although the first BAG was developed more than 40 years ago [10,18,19], an exploration into its potential for use in dental resin composites has only very recently begun. However, it is still unclear as to which type of and how many BAG fillers should be added in dental resin composites to fulfill the clinical requirement, i.e., relevant to the oral environment.

Although a number of recent review studies have provided critical analyses of the application of BAG in dentistry [20,21,22], these studies do not cover the main aspects of BAG-loaded RBCs and resin adhesives, i.e., the principal properties such as physicochemical properties, mechanical properties, mineralization ability, and *in vitro* biological responses. Thus, this narrative review aims to provide a comprehensive analysis of the existing literature, particularly focusing on BAG-loaded resin composites, by discussing the above-mentioned characteristics.

## 2. Methods

### 2.1. Research Question

Prior to the initiation of this narrative review, a research question was formulated: “Are BAG-loaded resin composites bioactive?” In this review, the definitions employed for establishing *in vitro* bioactivity in bioactive glass and the proposed mechanisms involved in these phenomena are used as blueprints for investigating whether BAG-loaded resin composites are bioactive.

### 2.2. Search Strategy

The electronic databases searched for identifying the relevant studies included Web of Science, PubMed, and Science Direct. The published scientific articles from January 2011 to January 2022 were assessed for this review. The keyword combinations of “bioactive glass OR bio-active glass OR bioglass” AND “resin composites OR resin OR resin adhesive” AND “dental OR tooth OR teeth” were used to search the relevant studies included in the electronic databases.

### 2.3. Inclusion and Exclusion Criteria

To maintain the focus of this review paper, the inclusion criteria for the review were the following (1) BAG-loaded dental restorative materials, including resin adhesive and filling restorative; (2) melt-derived BAG and sol–gel BAG, as they are common and easily prepared; (3) lab study: *in vitro* and *in vivo* study of dental resin composites. The exclusion criteria for the review were the following: (1) studies that involved BAG used for other applications such as varnishes, desensitizers, paste, glass ionomer, resin infiltrant, and air abrasion; (2) studies reporting commercial bioactive resin composites containing no BAG but showing bioactivity.

### 2.4. Searching

After searching, the title and abstracts of articles were assessed to be included or not based on the inclusion and exclusion criteria. Additional related references in the selected articles were checked and included in this review when meeting the inclusion criteria. All selected studies were imported into Endnote X9 software to be read and analyzed in detail.

## 3. Results

**Figure 2:**
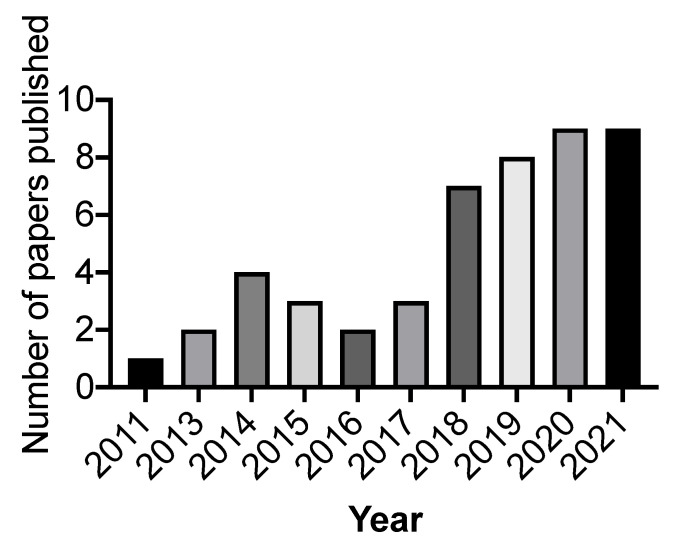
Number of papers published per year in the field of “bioactive glass-loaded resin composites”.

**Figure 3:**
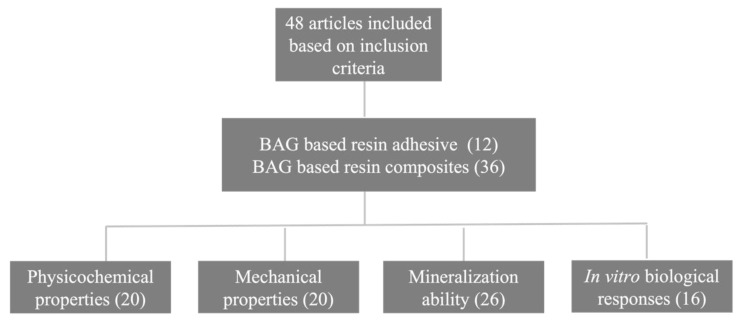
Flow chart of literature search showing the number of articles based on the characteristics of bioactive glass-loaded dental resins/resin composites.

The literature research returned a maximum number of papers (48) that met the inclusion criteria for the review and hence were chosen for the review. There has been heightened interest in the study of BAG-loaded resin composites over the last 10 years, as manifested by the gradually increasing number of publications in the field every year (Figure 2). The flow chart of the results of the literature search is presented in Figure 3. Among the studies, the physicochemical properties (20), mechanical properties (20), mineralization ability (26), and *in vitro* biological responses (16) of BAG-loaded resin composites have been investigated and summarized. In particular, limited studies have been conducted to explore the *in vitro* biological responses. It is worth noting that no *in vivo* study of BAG-loaded resin/resin composites was found. This is likely due to the difficulties in evaluating *in vivo* remineralization. Thus, methods should be explored for assessing *in vivo* remineralization of BAG-containing dental resin composites.

### 3.1. The Physicochemical Properties of BAG-Loaded Dental Resin Composites

The results show that a total of 20 studies [23,24,25,26,27,28,29,30,31,32,33,34,35,36,37,38,39,40,41,42] are related to the physicochemical properties of BAG-loaded dental resin composites. These researches highlight the degree of conversion (DC), depth of cure (DoC), light transmittance, polymerization shrinkage, water sorption (WS), water solubility (WL), and colour stability of experimental resin composites containing BAG fillers (Table 1).

#### 3.1.1. Curing Potentials (DC, DoC, Light Transmittance, Polymerization Shrinkage)

Experimental bisphenol A glycidyl methacrylate (bis-GMA)/triethylene glycol dimethacrylate (TEGDMA)-based resin composites with a total filler load of 70 wt% and variable amounts of 45S5 BAG (0, 5, 10, 20, and 40 wt%) have been investigated in their curing potential [27,35] and polymerization kinetics [40]. These studies indicated that BAG filler could diminish the DC [27,35], DoC [27], and light transmittance [27], as well as polymerization rate [40]. In Par et al.’s study [27], the DC and DoC were decreased after adding 40 wt% of BAG in resin composites. Furthermore, the decline in DC with a higher BAG loading occurred regardless of light transmittance, which was impaired by BAG fillers, but not in a dose-dependent manner. The direct inhibition of BAG on the curing potential of resin composites was further verified to be irreversible rather than simply retardation of the polymerization reaction. This was based on the fact that the post-cure reaction at 150 °C was unable to compensate for the initially decreased DC when using the bis-GMA and ethoxylated bisphenol A dimethacrylate (bis-EMA) resin systems [28]. Furthermore, the findings revealed that the DoC of all experimental materials was higher than the stipulated 1 mm established by ISO 4049 [43]. However, according to the “80% of max DC” method, BAG-20 and BAG-40 showed a DoC below 2 mm when cured for 20 s [27]. Their other research also found that the higher BAG amount diminished 24-h DC [28], the polymerization rate, and the 5-min real-time DC [40].

The curing potential of BAG-loaded resin composites based on different resin systems was also evaluated. 40 wt% BAG-loaded resin composites led to DC values of 35%, ~60%, and ~80% for bis-EMA, bis-GMA, and UDMA resin systems, respectively [28]. The DC value of UDMA-based resin composites was comparable to that of commercial composites (78–88%). In addition, the UDMA series also presented better light transmittance and a higher DoC than the bis-GMA and bis-EMA resin systems [26]. This was in good agreement with Nicolae et al. [30], which reflected no detrimental effects of BAG on UDMA/TEGDMA-based resin composites. Nevertheless, the addition of up to 20 wt% BAG significantly increased the DC in the bis-GMA/TEGDMA resin system. 

In addition, they also evaluated a new customized CaF_2_-containing BAG with a lower Na_2_O content (10.5 wt%) than that in the conventional 45S5 BAG (24.5 wt%) to functionalize bis-GMA/TEGDMA-based resin composites (Table 2) [36,41]. Contrary to the conventional 45S5 BAG, no negative effects on DC [36] and 5-min real-time DC [41] were identified in the resin composites incorporated with F-containing BAG. The addition of higher amounts (20 and 40 wt%) of F-containing BAG in resin composites significantly improved the DC of resin composites. It is worth noting that the F-containing BAG-loaded resin composites showed higher light transmittance than BAG-loaded resin composites for a given BAG amount [41]. In another study [42], F-containing BAG led to the increase in linear shrinkage and shrinkage stress of resin composites, contrary to conventional BAG, which shows a decrease in linear shrinkage and shrinkage stress due to the decrease in the degree of conversion. Moreover, except for 40 wt% F-containing BAG-loaded composites showing significantly higher shrinkage stress, other experimental composites containing BAG showed shrinkage stress values similar to the control composite.

The modified BAG with other ion doping was also reported [32]. These studies revealed that the incorporation of 10 wt% Cu-doped mesoporous bioactive glass (Cu-MBG) contributed to the highest DC of all the tested materials because of the favorable resin viscosity [32]. Bismuth-oxide-modified BAG [31] and niobium-doped BAG (BAGNb) [37] showed no effect on the DC. Furthermore, 21 days of immersion in phosphate buffer saline (PBS) decreased the DC values of all composites, while that of the 20 wt% bismuth-oxide-modified BAG group was significantly higher than that of both the 10 wt% bismuth-oxide-modified BAG group and the control resin composites [31]. Additionally, BAGNb showed higher hardness and softening in the solvent than in the BAG group [37]. The resin composite containing 10 wt% of silver-doped bioactive glass (Ag-BAG) showed a significantly small DoC at 0.76 ± 0.02, which was below the 0.8 acceptable ratio value [38]. The incorporation of 20 wt% BAG in commercial bulk-fill composite materials (SDR, Venus Bulk Fill, Filtek Bulk Fill) was also explored [34]. The findings demonstrated that 20 wt% BAG incorporation had no influence on the DC for Venus Bulk Fill and Filtek Bulk Fill, except for SDR, which showed a decreased DC. 

The effects of particle sizes (nanometric 30–50 nm, micrometric 5.6 μm, or hybrid) of the BAG fillers on the curing potentials were assessed after the addition of 15 wt% to a commercial flowable composite based on the bis-GMA/UDMA/TEGDMA system [39]. No significant difference in DC was found among the groups, regardless of the BAG particle sizes. Furthermore, the addition of BAG in resin composites decreased shrinkage stress independent of loading concentration compared with control groups containing no BAG. The effects of particle sizes were also reflected in another study [25], indicating that variations in the BAG particle size did not affect DC.

#### 3.1.2. Water Sorption, Water Solubility, and Colour Stability

The addition of 45S5 BAG fillers increased the wettability of the resin-based cement [29], which led to the increase in WS and WL of BAG-loaded resin composites [33]. In contrast, higher WS was found in biostable glass-loaded resin composites rather than in the S53P4 BAG-loaded composite group in Oral et al. study [24]. They also explored the impacts of silanization of glass fillers on WS and WL of resin composites and revealed that the higher WS and lower WL were found in the groups with silanized glass fillers. On the contrary, Elalmis et al. [33] found there to be both a decreased WS and WL after silanization treatment for BAG. Furthermore, the WS and WL of BAG and Al_2_O_3_-doped BAG-containing resin composites were not significantly different. The WS and WL further affected the colour stability of the material. This was conferred by the fact that a decrease in the colour stability of S53P4 BAG-loaded resin composites after immersion in staining solutions (tea, coffee, and water) for 1, 7, and 30 days was detected [23].

### 3.2. The Mechanical Properties of BAG-Loaded Dental Resin Composites

A total of 20 studies [24,25,29,30,32,33,34,35,38,44,45,46,47,48,49,50,51,52,53,54] were found to evaluate the mechanical properties of BAG-loaded dental resin composites, where flexural strength (FS), flexural modulus (FM), biaxial flexural strength (BFS), modulus of resilience (MR), fatigue crack growth, compressive strength (CS), microhardness (MH), bond strength, and fracture toughness have mainly been analyzed (Table 3).

Par et al. [35] evaluated the effects of 45S5 BAG on FS, FM, MR, and material reliability of resin composites. When the BAG amount was higher than 10 wt%, FS and MR were significantly reduced, while FM decreased even after adding 5 wt% BAG. Furthermore, materials with higher amounts of BAG yielded a higher degradation of FS, FM, and MR after artificial aging in water. This is in line with findings from other studies [29] which found a decrease in FS after the incorporation of 10 and 20 wt% of 45S5 BAG in the resin matrix. The finding contradicts one particular study [50], which demonstrated that the mechanical properties, such as flexural strength, fracture toughness, and fatigue crack growth of 0–15 wt% Na-free BAG-loaded resin composites, were superior to commercially available composite Heliomolar. Furthermore, no degradation in strength after 2 months of aging in bacteria media was shown in this study. The mechanical properties of resin composites containing BAG were also similar to or better than that of commercial composites, such as Filtek Z250 and Filtek Supreme Plus. 

Furthermore, a recent study revealed that the addition of S53P4 BAG fillers of less than 30 wt% did not affect FS and CS [46]. Han et al. [52] also presented that no adverse effects on FS and CS were found after the incorporation of 8, 16, and 23 wt% BAG in resin composites. Nicolae et al. [30] demonstrated significantly higher FS and FM values in bis-GMA/TEGDMA resin composites containing 20 wt% BAG fillers when compared with control composites containing no BAG fillers. However, no difference in FS and FM was found in UDMA/TEGDMA resin system when loaded with 20 wt% BAG fillers. It is noteworthy that the addition of more than 20 wt% BAG fillers caused a marked decrease in FS and FM in both resin systems. Furthermore, microhardness was increased in 20 wt% BAG-loaded Filtek Bulk Fill, while no changes were found in Venus Bulk Fill or SDR composite materials [34]. Their findings matched those of previous studies [29] which demonstrated an increase in MH with the addition of 10 and 20 wt% of 45S5 BAG. However, this was in contrast to other research [53] that reflected the negative impact of BAG on MH. Furthermore, particle sizes of BAG fillers showed no influence on MH [25]. 

In addition, the silanization of the inert and BAG particles was carried out using 2% of MPTS [24]. This did not reveal a significant effect on the FS and toughness of the composite. It has been reported that the polymerization of resin composites is not affected by the silanization using MPTS-silane [55]. In contrast, Elalmis et al. [33] showed that the silanization of BAG resulted in improved FS and FM while surface microhardness values decreased. Similarly, in another study [54], the silanized BAG-loaded composites exhibited significantly higher CS and FS than the non-silanized BAG composites. 

Other ion-doped BAG fillers may have a positive or negative influence on the mechanical characteristics of resin composites. The FS, FM, and MH of resin composites treated with alumina-doped 45S5 BAG were higher than those of the 45S5 BAG group [33]. In Marovic et al. study [32], 10 wt% Cu-MBGN-loaded resin composites showed lower FS than the BAG-containing composites and the BAG-free control group. Unlike FS, the FM and MH were enhanced by introducing Cu-MBGN fillers. When zinc-doped phosphate-based glass (Zn-PBG) was added to flowable resin composites [48], the FS was considerably lower than that in the control group. Nonetheless, all values surpassed 80 MPa, satisfying the ISO 4049 requirement [43] and matching or exceeding those of several commercially available flowable resins. The 45S5 BAG particle doped with 5, 10, and 15 wt% nanosilver resulted in reduced mechanical characteristics [51], whose FS values were slightly below the minimum flexural strength (80 MPa) recommended by ISO standard 4049 [43]. 

In addition to three-point bending flexural strength, research demonstrated that a decrease of 30% in the biaxial strength is observed for Ag-containing BAG-loaded composites [38]. In addition, adding Ag- or Zn-doped BAG [45], mesoporous bioactive glass (MBG) [44], or graphene oxide-doped bioactive glass (GO-BAG) [49] to the resin adhesive enhanced the microhardness. Furthermore, resin adhesive modified with Ag- [45,47] or Zn-doped BAG [45] showed no significant difference in shear bond strength, compared with control resin adhesive. However, 5% MBG-loaded resin showed a slight decrease in the bond strength [44]. The shear bond strength decreased as the amount of GO-BAG in the resins increased [49].

### 3.3. Mineralization Ability of BAG-Loaded Dental Resin Composites

A total of 26 pieces of research [14,15,25,33,36,37,44,45,47,48,49,52,53,54,56,57,58,59,60,61,62,63,64,65,66,67] are related to the mineralization ability of BAG-loaded dental resin composites, including the capacity of dental materials to release remineralizing ions, elevate surrounding pH, and precipitate apatite, as well as the abilities of anti-demineralization and remineralization of teeth and the maintenance of mechanical properties of teeth (Table 4). 

#### 3.3.1. The Release of Remineralizing Ions, pH Elevation, and Apatite Formation

In prior work, researchers created experimental orthodontic resins (bis-GMA/UDMA) with 5, 10, and 20 wt% 45S5 BAG [53]. It was discovered that resins containing 45S5 BAG had a high ability to alkalize the medium to ultimate pH values of around 10, regardless of the starting pH (i.e., 4 or 7). Based on the reactivity of BAG, the release of calcium and phosphate ions in both solutions with different starting pH was found to be correlated with an increase in pH. The group that released the most amount of calcium and phosphate ions was the one with 20 wt% 45S5 BAG. These findings were corroborated by the presence of a considerable volume of precipitates on the surfaces of BAG-containing resins after 28 days of storage in PBS. The impact of 15 wt% micro-sized (5.6 µm) and nano-sized (70 nm) 45S5 BAG on pH elevation and apatite production was examined by Odermatt et al. [25]. It was found that the nano-BAG composite caused a significantly quicker pH increase, to a final value of around 10.5 than the micro-BAG composite, which raised the pH to a final value of 8.5. In comparison to the micro-BAG composite, which had numerous dispersed apatite, the nano-BAG and hybrid-BAG composites created smaller and more uniformly distributed crystals.

High sodium levels in BAG can cause the dissolution of glass particles as well as glass swelling, followed by damage to the physical characteristics, durability, and lifespan of the composite resin. Moreover, reduced sodium levels in BAG would allow for the incorporation of additional beneficial components such as fluorine (F) [62,63], bismuth (Bi) [67], Zn [48], alumina (Al) [47,60], gallium (Ga) [64], niobium (Nb) [37], and Ag [47]. 

F-containing BAG-loaded composites resin with various loading amounts (0, 20, 40, 50, 60, and 80 wt%) have been created [62,63]. Their mineralization ability in terms of calcium, phosphate, and fluoride ion release, pH change [63], and apatite formation [62] have been reported when immersed in 10 mL of either Tris buffer (TB) with a pH of 7, or artificial saliva (AS) at a pH of 7 (AS7) or a pH of 4 (AS4). There was a rise in the surrounding pH, similar to previous studies, which was more apparent in resin composites containing higher BAG loading. Furthermore, the largest increase in pH (up to 3 pH levels) was found in the AS4 solution at the final time-point of 180 days. In terms of ion release, the initial quick rise in Ca concentration in TB might be attributed to the diffusion of Ca from the BAG to the less concentrated TB medium along a concentration gradient. The rise was minimal after it was equilibrated and not dependent on BAG loading. In the AS4 media, the effect of higher BAG loading was only distinct in the long term. There was an initial rise of PO_4_^3−^ concentration in both AS7 and AS4 media, which was not found in TB. The first rise in PO_4_^3−^ concentration was accompanied by a decline in F^−^, which might mean that fluorapatite (FAp), rather than HA, was initially created in AS. In another study [62], the apatite crystals produced on the disk surface in AS7 were strongly oriented and grew in orientation over time, comparable to human enamel. More crystalline apatite was found in AS4 immersion than in TB, while the latter showed no distinctive layer of precipitation on the disk. Al-Eesa et al. [54] further investigated the effects of silanization on the F^–^ release and apatite production of F-containing BAG-loaded resin composites. The fluoride concentrations for the silanized and non-silanized glass upon immersion in AS7 were very similar. The silanized and non-silanized BAG modified composites had identical fluoride concentrations in AS4 until the 28-day time point; however, the non-silanized composite released higher fluoride from 28 days onwards. That said, silanization had no effect on the production of apatite. 

Other experiments employing SBF with a pH of 4 and 7 found comparable pH changes in surrounding solutions of BAG-loaded resin composites [65]. In the SBF at pH of 4, a final pH increase of 2 was seen; however, in the noncariogenic SBF at pH 7, a pH increase of 0.25 was observed. Moreover, a rise in PO_4_^3−^ concentration was discovered in this study, but no change in F^−^ concentration was discovered. Using hydrochloric acid (HAS) (pH 2.6) and lactic acid (LAS) (pH 4.5), Par et al. [36] investigated the acid-neutralizing capabilities of resin composites incorporated with CaF_2_-containing BAG with a lower Na_2_O content (10.5 wt%) than that in the conventional 45S5 BAG (24.5 wt%). The experimental composites showed higher HAS acid-neutralizing capabilities with final pH values in the range of 2.9–9.6 compared to 9.2–10.1 for LAS in just over one hour. It is worth noting that the ultimate high alkaline pH values (8.2–9.6) of HAS were only achieved by the 20 wt% and 40 wt% BAG-containing resin composites, whereas the experimental composites with lesser BAG levels attained substantially lower pH values (2.9–6.9). This research differs from another study which showed a final pH of 9.2 in HAS using only 10 wt% nano-sized (30–50 nm) bismuth (Bi)-modified 45S5 BAG-doped resin composites [67]. 

Davis et al. [66] explored the ion release of resin composite containing BAG fillers with different Si contents (BAG 61 (61% Si; 31% Ca; 4% P; 3% F; 1% B) and BAG 81 (81% Si; 11% Ca; 4% P; 3% F;1% B)). As was evident in their results, significantly more fluoride ions were released from the BAG 81 composite after 222 h compared with BAG 61. On the contrary, the BAG 61 composites revealed more release of calcium ions during each of the 2- and 22-h time periods.

In addition to F-doped BAG, the resin composite containing Zn-doped phosphate-based bioactive glass (Zn-PBS) increasingly released more Ca, P, Zn, and Na ions with a rise in Zn-BAG concentration [48]. This phenomenon was also shown in gallium (Ga)-doped mesoporous bioactive glass (GaMBN)-modified resin composites, revealing the increased release of Ca, P, and Ga ions along with a decrease in pH with immersion time [64]. The Al-doped 45S5 BAG-containing resin composite showed decreased apatite formation after 28 days of immersion in SBF [33] compared with conventional 45S5 BAG. This might be because Al can stabilize the glass structure because of the elimination of non-bridging oxygen [68], even though no difference in WS and WL between these two BAGs has been mentioned before. In contrast, the incorporation of niobium-doped BAG (BAGNb) into experimental adhesive resins [37] produced higher mineral deposition than the control group and BAG-containing composites after 28 days in SBF. In addition, an apatite-like phase was also detected on resin composites containing Ag-doped BAG after 14 days of storage in SBF [47,60]. However, there was no comparison between apatite formation in Ag-doped BAG and BAG in this study. In addition to conventional SBF evaluation, the bacterial biofilm growing medium also leads to apatite formation on the surfaces of the BAG composites [58].

The commonly utilized analyses for apatite formation, such as scanning electron microscopy (SEM), Fourier-transform infrared spectroscopy (FTIR), and X-ray diffraction (XRD), as shown in the aforementioned research [54,62], could not completely identify the types of apatite generated due to the overlap of the HA and FAp peaks. A novel method, named ^31^P and ^19^F magic angle spinning-nuclear magnetic resonance (MAS-NMR), was used to compare the chemical shifts of the original form of phosphate and fluoride in the glass to the new phosphate and fluoride compounds produced to analyze apatite formation. This was carried out by analyzing the powder from the submerged disks in Tris buffer at a pH of 7.3 (TB); neutral artificial saliva at a pH of 7 (AS7); and acidic artificial saliva at a pH of 4 (AS4) [57]. All of the spectra of the disks revealed a peak of crystalline apatite between 2.8 and 3 ppm in the ^31^P MAS-NMR [57]. The loss of the wide amorphous signal orthophosphate peak from the original unreacted glass and the emergence of a small and sharp peak at 2.8 ppm in the BAG disks submerged in AS4 suggested that the majority of the BAG particles had reacted to create apatite. On the contrary, a combination of these two components was found in TB and AS7. Faster degradation of the BAG disk was found in acidic media AS4, which supported the previous findings on ion release, pH changes, and apatite formation in acidic solutions [62,63]. The FAp signal at roughly −103 ppm [69] after 6 h was found in all immersion solutions. This signal was strongest for disks in the AS4 medium, revealing that acidic media causes more glass to convert to FAp. It is crucial to highlight that less glass deteriorated in AS7. The signal in TB showed a peak at roughly −108 ppm, which was attributed to crystalline fluorite (CaF_2_) [69], other than the peak of FAp at −103 ppm. The former, on the other hand, was a little sharper and more vivid than the latter. 

Furthermore, a novel scanning electrochemical microscopy (SECM) analytic approach [59] was utilized to measure the changes in Ca^2+^ concentration and pH at a distance of 20–1000 µm above the BAG-containing composites. In contrast to traditional assays for detecting ion concentration and pH, SECM [70] is a real-time non-invasive technology that permits accurate (within 10 µm) placement of the electrochemical probe above the surface of the substrate without touching or destroying it. The increase in local concentration (at z = 20 µm) for BAG particle sizes of 125–150, 75–90, 38–45, 38, and 5 µm was 100, 88, 166, 213, and 225 µM, respectively. However, none of the BAG-containing composites showed more than a 0.2-unit pH shift, indicating that the quantity of BAG in the resin composite was inadequate to release enough OH^−^ to change the local pH. Furthermore, the ion concentration and pH above the composites declined as one moved away from it. According to these findings, BAG-loaded resin composites may be more advantageous for mineralization at close range. 

#### 3.3.2. Anti-Demineralization and Remineralization of Enamel and Dentin

It has been reported that 45S5 BAG-loaded resin composites demonstrated more mineralized layer occluding dentin tubules after immersion in SBF for 21 days [52]. Moreover, resin composites incorporated with 65S BAG (65% Si, 31% Ca, and 4% P in mol%) were used for the remineralization of the demineralized dentin when placed at a gap of 50 μm between each other [14]. After 2 weeks of storage in SBF and PBS, the dentine surface was blocked by mineral components together with an increase in the microhardness of the dentin. Another study used F-containing phosphate-rich BAG (BAG-F)-doped resin composites demonstrating a stronger ability to remineralize the demineralized dentin after extended AS storage for 30 days compared to the BAG-loaded group [15]. Furthermore, following 30 days of remineralization, the specimens exposed to BAG-F had the greatest increase in modulus of elasticity. The BAG-F group also induced greater enzyme inhibition on cathepsin K (CTP) (3 d) and matrix metalloproteinases (MMP) (30 d) compared to the BAG group.

The preceding investigation only revealed the remineralization benefits of BAG-loaded restorative materials confined to the dental tissues directly next to them. One study [56] dealt with the experimental resin composites incorporated with conventional 45S5 BAG and low-Na F-containing BAG, which were placed at a standardized distance (5 mm) with human enamel blocks. The maintenance of enamel microhardness was evaluated through immersion in a repeatedly replenished lactic acid solution. A 20 wt% of BAG-loaded resin composites maintained the initial enamel microhardness for up to 5 acid addition cycles (20 d). On the other hand, the group with 20 wt% F-containing BAG was only able to keep the original enamel microhardness for three acid cycles (12 d), which was also found in the 10 wt% BAG group. Moreover, the 10 wt% F-containing BAG group could only maintain original enamel microhardness for two acid addition cycles (8 d). This is consistent with the more notable alkalization ability of the 20 wt% BAG group, which could maintain a plateau at pH = 9–10. The 10 wt% BAG and 20 wt% F-containing BAG-loaded resin composites showed only transient alkalization, followed by a drop in pH values. 

Par et al. [61] used the same experimental approach to investigate whether dentin could also benefit from the remote-acting protection of BAG-containing resin composites against demineralization. Dentine microhardness was maintained over different numbers of acid additions, as follows: 20 wt% BAG (up to 12 days), and 10 wt% BAG, and 20 wt% F-containing BAG (up to 4 days). Except for the 20 wt% BAG group, which plateaued at pH = 9, other materials attained a stable pH at only 6–7. Moreover, the remineralization and the anti-demineralization length of enamel were tested using micro-computed tomography (micro-CT) by submerging samples in demineralization solution for 6 h, which was followed by 18 h in remineralization solution for 14 days [44,45,49,64]. Results showed that as the BAG concentration increased, so did the remineralization and anti-demineralization length.

### 3.4. In Vitro Biological Responses of BAG-Loaded Dental Resin Composites

To assess the *in vitro* biological responses of BAG-loaded dental resin composites, a total of 16 studies [29,37,38,44,45,46,47,48,49,52,53,59,60,64,71,72] analyzed the *in vitro* biocompatibility and antibacterial effects of this experimental composite (Table 5). 

#### 3.4.1. *In Vitro* Biocompatibility of BAG-Loaded Resin Composites

Previous research [72] determined the *in vitro* biological responses of dental resin composites containing 0, 5, 10, and 15 wt% of BAG65 (65% SiO_2_, 31% CaO, 4% P_2_O_5_ in mol%) and BAG61 (3 mol% F added), using undifferentiated dental pulp cells (OD-21). The 7-d culture media extracts from resin composites with a dilution level of 1:8 or higher dramatically impaired cell viability. Direct exposure to newly cured BAG-loaded composites reduced cell viability as well, with no statistical difference in cell viability between BAG-loaded resin composites. However, after preincubation in a cell culture medium for 7 days, the experimental composites showed similar cell metabolic activity to the control. 

On the contrary, no difference in human osteoblast (MG63) viability was found between the control group without composites and the experimental groups containing BAG-loaded resin composites when using the direct-contact cell model in another study [29]. Another investigation found that 8 wt% 45S5 BAG-loaded resin composites were not cytotoxic to mouse fibroblast L929, while 16 wt% and 23 wt% BAG groups did reveal cytotoxicity [52]. It is interesting to note that the cell viability was returned to normal after adjusting the elevated pH value of the extracts to neutral. 

When doping other metal ions in BAG, the cell viability may be affected. Experimental adhesives containing 2 wt% BAG or 2 wt% niobium-doped BAG (BAGNb) reached levels of 126.89% and 129.53% of cell viability, respectively, compared with the control resin adhesive containing no BAG or BAGNb fillers [37]. Similarly, 5 wt% gallium-doped mesoporous bioactive glass (GaMBN)-containing resin adhesive revealed significantly higher viability of human dental pulp stem cells (hDPSCs) than other groups after 24 h of cell culture [64]. Furthermore, BAG and graphene oxide (GO)-combined BAG had no effects on the cell viability of human gingival fibroblasts (HGF-1) using the direct-contact cell model compared with the control groups without experimental resin adhesives [49]. 

#### 3.4.2. *In Vitro* Antibacterial Effects of BAG-Loaded Resin Composites

The previous study [46] evaluated the antimicrobial efficacy of dental resin composites containing 5, 10, and 30 wt% of microparticulate S53P4 BAG. The viability of *Escherichia coli* (*E. coli*), *Staphylococcus aureus* (*S. aureus*), and *Streptococcus mutans* (*S. mutans*) was decreased significantly after exposure to extracts from BAG-loaded resin composites in a dose-dependent manner. In terms of anti-biofilm capability, BAG loading can result in a 0.5 log decrease in the viable count of *S. mutans*. This is in line with the findings of other studies [53,59]. Here, the resins containing 5, 10, and 20 wt% 45S5 BAG demonstrated a significant decrease in *S. mutans* viability, regardless of BAG levels [53]. Another study revealed that a dose-dependent decrease in the viable colonies of *S. mutans* was observed when using the extracts of 8, 16, and 23 wt% 45S5 BAG-loaded composite resins [52]. Furthermore, the typical and well-ordered chain-like arrangement of *S. mutans* was lost after adhesion on the surface of the BAG-loaded composite resins.

The antibacterial potential of BAG-loaded resin composites was also manifested by a lower average depth of bacterial penetration (61%) into the marginal gap in comparison to the control group, which showed 100% penetration [71]. In Aponso et al. study [59], the BAG composites failed to cause a substantial shift in pH while still demonstrating a reduced height and volume in *S. mutans* biofilm growth. Moreover, the addition of only 5 wt% mesoporous BAG nanoparticles could also show a significant antibacterial effect on both *S. mutans* and *Porphyromonas gingivalis* (*P. gingivalis*) [44] attributed to the large specific surface area.

To further promote the antibacterial properties of BAG-loaded resin composites, the most straightforward technique is doping additional antimicrobial ions into BAG. Ag was selected as the dopant because it possesses antibacterial properties [73]. The addition of 5, 10, and 15 wt% Ag-doped 58S BAG (Ag-BAG) in resin composites demonstrated improved antibacterial potentials against *S. mutans*, *E. coli*, and *Lactobacillus casei* (*L. casei*) compared with the control resin composites without Ag-BAG [38,47,60]. The composite extracts from 8 days [60] and 22 days [38,47] of immersion in PBS significantly inhibited *S. mutans* growth. The number of *E. coli* and *S. mutans* bacteria adhered to the resin composites was significantly decreased after loading with 15 wt% Ag-BAG. Moreover, the number of dead bacteria on 10 wt% or more of Ag-BAG-loaded resin composites was much larger, according to the quantitative analysis of the confocal laser scanning microscopy (CLSM) pictures. 

The resin composites incorporated with Zn-PBG also revealed the antibacterial activity against *S. mutans* [45,48]. This antibacterial property is thought to be attributed to the bacterial resistance of Zn ions [74,75], and Zn-PBG has been shown to have antibacterial potential [76]. At the same time, resin composites containing a mixture of 5 wt% GO combined with BAG [49] had a considerably stronger antibacterial impact on *S. mutans* due to the damage to the cell membrane and the metabolic activity caused by GO [77]. However, there was no significant difference in the viability of *S. mutans* on resin adhesive containing Ga-doped mesoporous bioactive glass nanoparticles (GaMBN) [64]. 

## 4. Discussion

### 4.1. The Effects of BAG Fillers on Physicochemical and Mechanical Properties of Resin Composites

When adding unsilanized BAG fillers in resin composites, one crucial issue to consider is that BAG fillers may not be well bonded to the composite matrix, resulting in unsuitably low curing potential and mechanical properties. The potential of BAG to adversely affect the curing potential (DC, light transmittance, and DoC) was reported [27] and was highly dependent on the resin system [26,28] in the following order: bis-EMA > bis-GMA > UDMA. This is surprising because the replacement of small barium fillers (1 µm) and silica fillers (12 nm) with larger BAG fillers (4 µm) [27] reduces the resin/filler interfacial surface area, which leads to the higher resin mobility [78]. Furthermore, the lack of a silane coating on BAG fillers was also expected to reduce the bond quality between the fillers and the resin matrix, which also caused the lower resin viscosity. The resin with high mobility and low viscosity should point to a higher DC [78,79], which contradicts the decreased DC values after the incorporation of small BAG fillers, as shown in Par et al. study [27]. This implies that unsilanized BAG fillers may have a direct inhibition effect on the curing potential of resin composites. The direct inhibition of unsilanized BAG fillers on the polymerization reaction is likely to be a result of the inactivation of free radicals resulting from electron transfer to oxides on the surface of the BAG particles. Nevertheless, in the Nicolae et al. study [30], adding up to 20 wt% BAG significantly increased the DC in the bis-GMA/TEGDMA resin system. This contradicted the aforementioned study [27,28], which showed that the DC declined with a higher amount of BAG having a particle size of 4 µm. The increased DC values found in Nicolae et al. study [30] may result from the high resin mobility due to the comparatively larger particle size (50 µm) of the BAG fillers. The high resin mobility may outweigh the free radical inhibition induced by the BAG and consequently yields a high DC. 

**Figure 4:**
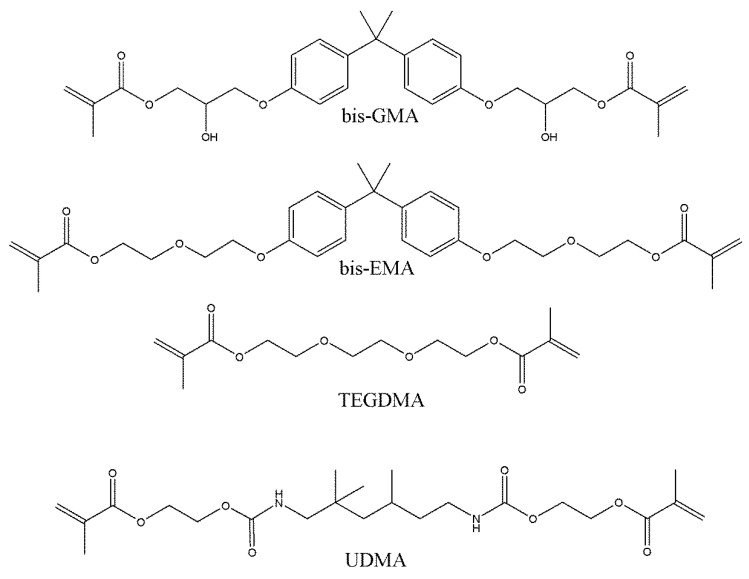
Chemical structure of different monomers used in resin-based dental composites.

In contrast, the DC in the UDMA resin system remained unimpaired by the increasing BAG amounts, which was attributed to its distinct capability to polymerize through -NH- groups (Figure 4) [80]. This can make the UDMA resin system less susceptible to the inhibition of the curing potential caused by the unsilanized BAG fillers. In addition, the UDMA series also presented better light transmittance and a higher DoC than the bis-GMA and bis-EMA resin systems [26]. This was in good agreement with Nicolae et al. [30], which reflected no detrimental effects of BAG on UDMA/TEGDMA-based resin composites. In addition to conventional 45S5 BAG, the above research implied that ion-doped BAG had no negative effect on the DC even in the bis-GMA resin system, which might be because of the large interfacial area between the fillers and the resin matrix caused by the disruption of the silica network due to the ion doping. 

The decreased DC corresponded to the impaired mechanical properties after adding more than 10 wt% BAG in resin composites [27]. However, the mechanical properties of experimental composites with up to 20 wt% of BAG still fulfilled the minimum FS value of 80 MPa required by ISO 4049 [43]. Furthermore, a recent study revealed that the addition of S53P4 BAG fillers of less than 30 wt% did not affect FS and CS [46]. The above findings revealed that low amounts of BAG did not impair the mechanical properties of resin composites. However, this is in contradiction to the reported findings by Oral et al. [24], demonstrating that 3 wt% BAG-loaded composites showed significantly lower FS, FM, and toughness. The difference may be dependent on the different particle sizes of BAG fillers. Oral et al. used BAG fillers with larger particle sizes of 315–1000 μm [24] compared to the other research, which used smaller particle sizes of 50 μm [30], 0.04–3.0 μm [50], 0.6 μm [46], and 7.26 μm [52], respectively. The fillers with small particle sizes show a high surface, which consequently leads to high surface energy at the interface between fillers and resin matrix and improvement in the mechanical properties [81]. Furthermore, ion-doped BAG fillers showed negative impacts on the mechanical properties, which may be explained by the disruption of the silica network, which results in poor bond quality between the glass fillers and the resin matrix. 

The BAG-induced polymerization inhibition can be partly responsible for other performances, such as the WS and WL [24,33,82]. Water sorption of resin composites is a diffusion-controlled process and lasts until the resin is fully saturated with water [83]. Soluble components, such as unreacted monomers and leaching ions, are eluted from the composite along with the water uptake. Increasing the ratio of the BAG fillers in the resin composites demonstrated a distinct effect on WS: 40 wt% BAG-loaded composites showed almost six times higher WS than control composites containing no BAG, which is due to the high wettability and hydrophilicity of the BAG-loaded composites. The higher WS found in silanized BAG fillers in the Oral et al. [24] study may be due to the polysiloxane networks caused by silanization, which renders the diffusion of water into the polymer matrix. Moreover, the low WL in the silanized glass filler groups can be explained by the protection of the glass structure by the silane coating. When silane groups are linked to inorganic fillers, their hydrophobicity decreases the interaction between water and the filler, thus delaying its dissolution [84,85]. 

Overall, BAG fillers had no effects on the curing potential and polymerization of the UDMA-based resin composite compared to bis-GMA- and bis-EMA-based resin composites. Furthermore, the BAG-induced inhibition may cause impaired mechanical performances. However, the mechanical properties of resin composites with no more than 20 wt% BAG loading and of small particle sizes were comparable to those containing only reinforcing fillers and commercial resin composites. 

### 4.2. The Effects of BAG Fillers on the Mineralization Ability of Dental Resin Composites

It has been reported that resins containing 45S5 BAG had a high ability to alkalize the medium to ultimate pH values of around 10, together with the release of calcium and phosphate ions [53]. These findings were corroborated by the presence of a considerable volume of precipitates on the surfaces of BAG-containing resins after 28 days of storage in PBS. This mineralization ability of BAG-loaded resin composites results from the dissolution and bioactivity of BAG. The underlying mechanisms that enable BAG to be bioactive have been discussed in detail [9,10,86]. When it comes into touch with bodily fluids, BAG immediately undergoes dissolution and degradation via the release of Na^+^ and Ca^2+^ from the glass surface into the surrounding. At the same time, H^+^ from the surrounding solution is exchanged, causing the elevation of pH. This results in the formation of silanol groups (Si–O–H). With the increase in surrounding pH, the silica network is further degraded, leading to the formation of a negatively charged gel which is composed of orthosilicic acid (Si(OH)_4_). This negative silica gel surface layer attracts Ca^2+^ and PO_4_^3−^ and promotes the formation of a layer of amorphous calcium phosphate (ACP), which further crystallizes into carbonated hydroxyapatite (CHA). In dentistry, the formed apatite could occlude the dentin tubules and provide anti-demineralization and remineralization ability [52,56,61], which was beneficial for preventing secondary caries.

Furthermore, the released Ca^2+^ concentration increased with the increase in BAG loading in resin composites in a dose-dependent manner [53]. This can be explained by the diffusion of Ca from the higher BAG contents to the less concentrated medium along a higher concentration gradient. Furthermore, the stronger acid neutralization ability found in the nano-BAG composite [25] could be explained by the 30 times more specific surface area of nano-sized BAG particles available for ion exchange. The more released ions due to the larger surface area of BAG particles were also reflected in another study [66], which demonstrated that more ions were released from the BAG-composite containing BAG fillers with the larger surface area (316 m^2^ g^−1^) compared with those with the small surface area of 129 m^2^ g^−1^.

In addition to the conventional BAG-loaded resin composites, F-containing BAG integrated into RBCs is also reactive in terms of pH elevation [63], ion release [63], and apatite formation [62] in various solutions (TB, AS7, and AS4). In particular, the greatest pH rise was observed in acid solution AS4, which benefits clinical applications since the acid attack caused by cariogenic bacteria can be neutralized by the dissolution of BAG. Furthermore, the quick and high ion release from BAG-loaded resin composites in an acidic solution could produce a thicker apatite layer on the disk’s surface [62]. This may provide benefits for the antibacterial potential and aid in the prevention of teeth demineralization and recurrent caries. In contrast, the relatively small increase in ion concentration and pH in AS7 indicated that BAG-loaded resin composites were stable in neutral media. 

The above findings indicated that BAG-loaded resin composites could be considered smart bioactive materials, as evidenced by the fact in other research [36,65,66]. Both BAG 61 (61% Si; 31% Ca; 4% P; 3% F; 1% B) and BAG 81 (81% Si; 11% Ca; 4% P; 3% F;1% B)) were capable of recharging fluoride [66]. This implied that F-doped BAG-containing resin composites might be sufficient for the long-term prevention of caries through repeated calcium and fluoride release and recharge. Another experiment [65] employed SBF with a pH of 4 and 7 and showed a final pH increase of 2 in the SBF at a pH of 4, however, a pH increase of 0.25 in the noncariogenic SBF at pH 7. This suggested that the pH shift was related to the original pH of the solutions. Hence, these BAG-loaded composites could be regarded as smart materials which behave differently in the different media relative to oral environment. In addition, the comparison of acid-neutralizing capabilities of BAG-loaded resin composites in two kinds of acid, including hydrochloric acid (HAS) (pH 2.6) and lactic acid (LAS) (pH 4.5), to simulate an acid attack by hydrochloric acid from gastric acid and lactic acid attack produced by biofilm bacteria, was conducted [36]. Experimental composites demonstrated higher acid-neutralizing capabilities in HAS than those in LAS. Only resin composites containing 20 wt% and 40 wt% BAG produced high alkaline pH values (8.2–9.6) in HAS. However, anotehr research [67] showed a final pH of 9.2 in HAS using only 10 wt% nano-sized (30–50 nm) Bi-modified 45S5 BAG-doped resin composites. This might be attributed to the nano-sized BAG, which has a larger reactive surface area than the micro-sized BAG (3 µm) in Par et al. study [36]. This could give rise to problems in terms of the quick degradation rate of the bioactive fillers [87] as well as H_2_O pick up from the atmosphere [88]. However, the LAS acid-neutralization capability with a final pH of 9.8 was identical in both studies, suggesting that the influence of BAG particle sizes becomes more significant in higher acidity solutions (HAS) than in LAS. 

Incorporating other ions in BAG demonstrated diverse mineralization results. The Al-doped 45S5 BAG-containing resin composite showed decreased apatite formation [33] compared with conventional 45S5 BAG. This might be because Al can stabilize the glass structure because of the elimination of non-bridging oxygen [68], even though no difference in WS and WL between these two BAGs has been mentioned before. However, higher mineral deposition produced by adhesive resins containing niobium-doped BAG (BAGNb) [37] can be attributed to the higher degree of softening of BAGNb than the BAG group, as shown in the study. Moreover, niobium has been researched as a component of many biomaterials and has been shown to increase mineral deposition [89,90,91]. 

To summarize, both the conventional BAG and F-containing BAG integrated into RBCs, considered to be smart materials in a dose-dependent manner, are reactive in terms of ion release, pH elevation, and apatite formation in various solutions. This aids in the anti-demineralization and remineralization of enamel and dentin and could prevent recurrent caries. Furthermore, incorporating additional beneficial ions in conventional BAG could provide a mineral reservoir and allow for special application. 

### 4.3. The Effects of BAG Fillers on In Vitro Biological Responses of Dental Resin Composite

When BAG-loaded resin composites meet biological fluids, it is important to consider the potential cytotoxicity due to the release of residual monomers [92] resulting from an incomplete polymerization reaction [72,93]. Furthermore, the undesired changes in localized pH and ion concentration can occur during the interaction of the BAG fillers with the surrounding solutions, which may also have cytotoxicity. At the same time, biocompatibility should be explored in detail to estimate whether BAG-loaded resin composites are bioactive or not. 

Common *in vitro* biocompatibility tests have included cell viability using culture media extracts from resin composites and direct exposure to BAG-loaded composites, which demonstrated the impaired cell viability [52,72]. However, the effects of BAG loading on this cytotoxicity behaviour differed in different research. In Salehi et al. study [72], no statistical difference in cell viability between BAG-composites groups indicated that cytotoxicity is primarily attributed to the release of residual monomers, not from the leached ions and raised pH due to the dissolution of the BAG fillers. On the contrary, in Han et al.’s study [52], only resin composites containing 16 wt% and 23 wt% BAG revealed decreased cell viability, which returned to normal after adjusting the elevated pH value of the extracts to neutral. This implied that the cytotoxicity of BAG-loaded resin composites was also attributed to the raised pH resulting from the dissolution of BAG in a dose-related way. However, this non-realistic pH change may not be observed *in vivo* [94,95] due to *in vivo* dynamic conditions that cause a continuous dilution of the leached ions [96]. Therefore, a variety of preconditioning solutions, such as simulated body fluid (SBF) [97,98,99,100], alpha minimum essential medium (α-MEM) [101,102], and tissue culture medium (TE) [96,103] have been put forward over the years to test BAG materials in cell culture. Research has shown that after preincubation in a cell culture medium for 7 days, the experimental BAG-loaded composites showed similar cell metabolic activity to the control. Thus, BAG-loaded dental composites may not provide chronic cytotoxic effects *in vivo* since the relatively short-term exposure to body fluids will lead to the extraction of “toxic” components, including residual monomers, released ions, and elevated pH. 

On the contrary, there was research [29] showing that no negative influence on human osteoblast (MG63) viability was found in BAG-loaded resin composites. This could be due to the increased surface microhardness after the addition of 10 and 20 wt% of 45S5 BAG, as previously stated [29], which may result in less degradation and solubility of composites. Furthermore, the difference in cell viability might be attributed to the used cell types. For the *in vitro* biocompatibility test of BAG-loaded resin composites, different cells were assessed, including undifferentiated dental pulp cells (OD-21) [72], human osteoblast (MG63) [29], human dental pulp stem cells (hDPSCs) [64], human gingival fibroblasts (HGF-1) [49], mouse fibroblasts (L929 cells) [52], and primary pulp fibroblasts [37]. MC3T3-E1 cells have been utilized in several studies [104,105,106] to assess the cytotoxicity of resin composites but have never been used to evaluate the cell viability of BAG-loaded resin composites. Furthermore, limited reports are available on the cells associated with bone formation. Therefore, it is essential to evaluate the impact of BAG-loaded resin composites on MC3T3-E1 cellular viability to assess whether these experimental composites could induce cell mineralization. 

Compared with other restorative materials, resin composites are more prone to accumulate bacteria on the surface during early plaque development, which could cause secondary caries. Therefore, the development of resin composites possessing an antibacterial effect is an ongoing avenue of research. The previous research [46,52,53] confirmed the inhibition efficacy of resin composites containing BAG fillers on the viability of *E. coli*, *S. aureus*, *S. mutans*, and *P. gingivalis*, which was mainly due to the release of calcium and phosphate ions and pH elevation resulting from the dissolution of BAG fillers in resin composites. The mechanisms of the antibacterial effect of BAG have been discussed in two aspects [13]: one is the alkaline environment caused by the release of OH^−^ because of the dissolution of BAG; the other is the presence of irregular BAG debris that is needle-like, which can damage the cell wall. These could also explain the antibacterial effects of BAG-loaded resin composites. However, it would be more appropriate to explore the antibacterial properties of BAG-loaded resin composites after preconditioning treatment to better simulate *in vivo* conditions.

To further promote the antibacterial properties of BAG-loaded resin composites, the most straightforward technique is doping additional antimicrobial ions into BAG. The resin composites incorporated with Ag-doped 58S BAG (Ag-BAG) [38,47,60], Zn-PBG [45,48], and GO combined with BAG [49] also revealed the antibacterial activity. Ag was selected as the dopant because it possesses antibacterial properties [73]. However, the pH value in the surrounding solution remained neutral [60], confirming that the reported bactericidal activity is mainly caused by the leached Ag ions and not pH variations. In addition, aesthetic issues of silver oxidation may prevent the use of Ag-BAG-containing resin composites in anterior teeth. Furthermore, the cytotoxicity of Ag-BAG-containing resin composites should be taken into account in future research. 

In summary, BAG-loaded resin composites may induce cytotoxicity, which is mainly dependent on BAG concentration and the types of cells used. Furthermore, preincubation of these resin composites in media prior to the cell study showed unaffected cell metabolic activity. In addition, BAG-loaded resin composites demonstrated antibacterial properties, which are beneficial for the prevention of secondary caries. 

## 5. Conclusions

This review confirms that BAG-loaded resin composites can be regarded as bioactive materials, which is manifested by the *in vitro* antibacterial performance on cell viability and biofilm formation. Furthermore, BAG-loaded resin composites exhibit excellent mineralization ability reflecting enhanced ion release, pH elevation, and apatite formation, especially regarding high BAG loading. This aids the anti-demineralization and remineralization of teeth. However, for the improvement in the mineralization ability of resin composites through the doping of a high concentration of BAG fillers, more than 20 wt%, may come at the cost of other properties, such as the curing potential and mechanical properties, due to the direct inhibition of unsilanized BAG fillers on polymerization of bis-GMA-based resins. 

In summary, most previous studies dealing with the BAG-loaded resin composites only focused on one or some aspects using different resin systems, BAG types and BAG amounts. Therefore, a direct comparison between results from different studies may be biased. As such, it is essential to find an optimal balance between mechanical properties, mineralization ability, and *in vitro* biological responses of BAG-loaded resin composites.

## Figures and Tables

**Table 1:** The research conducted on physicochemical properties of BAG-loaded dental resin composites.

Research	Main Findings
[23]	S53P4 BAG led to a decrease in the colour stability of resin composites after immersion in staining solutions (tea, coffee, and water) for 1, 7, and 30 days.
[24]	A higher WS was found in biostable glass-loaded resin composites rather than in the S53P4 BAG-loaded composites.
[25]	Variations in the BAG particle size did not affect DC.
[26]	The UDMA series presented better light transmittance and a higher DoC than the bis-GMA and bis-EMA resin systems.
[27]	The addition of 40 wt% of BAG led to the decrease in DC and DoC. Light transmittance was impaired by BAG fillers not in a dose-dependent manner.
[28]	BAG incorporation in resin composites diminished 24-h DC. Post-cure reaction at 150 °C was unable to compensate for the initially decreased DC.
[29]	The addition of 45S5 BAG fillers increased the wettability of the resin-based cement.
[30]	BAG fillers reflected no detrimental effects on the DC of UDMA/TEGDMA-based resin composites.
[31]	Bismuth-oxide-modified BAG showed no effect on the DC.
[32]	The 10 wt% Cu-doped mesoporous bioactive glass contributed to the high DC.
[33]	45S5 BAG fillers led to the increase in WS and WL of BAG-loaded resin composites.
[34]	A 20 wt% BAG incorporation had no influence on the DC for commercial composite Venus Bulk Fill and Filtek Bulk Fill, except for SDR, which showed a decreased DC.
[35]	BAG filler could diminish the DC.
[36]	F-containing BAG showed no negative effects on DC.
[37]	Niobium-doped BAG showed no effect on the DC, and higher hardness and softening in the solvent than in the BAG group.
[38]	The 10 wt% of silver-doped bioactive glass showed a significantly small DoC at 0.76 ± 0.02 mm.
[39]	BAG particle sizes showed no significant effects in DC.
[40]	BAG fillers diminished polymerization rate and the 5-min real-time DC.
[41]	F-containing BAG showed no negative effects on DC and higher light transmittance than conventional BAG.
[42]	F-containing BAG led to the increase in linear shrinkage and shrinkage stress of resin composites, contrary to conventional BAG.

**Table 2:** The comparison of compositional details of conventional 45S5 BAG and CaF_2_-containing BAG.

Compositions	Contents (wt%) in Different BAG	
	45S5 BAG	CaF_2_-Containing BAG
SiO_2_	45.0	33.5
CaO	24.5	33.0
Na_2_O	24.5	10.5
P_2_O_5_	6.0	11.0
CaF_2_	0	12.0

**Table 3:** The research conducted on mechanical properties of BAG-loaded dental resin composites.

Research	Main Findings
Oral et al. [24]	The silanization of BAG particles did not reveal a significant effect on the FS and toughness of the composite.
Odermatt et al. [25]	Particle sizes of BAG fillers showed no influence on MH.
Costa Lima Assad et al. [29]	A 10 and 20 wt% of 45S5 BAG showed a decrease in FS and an increase in MH.
Nicolae et al. [30]	The 20 wt% BAG fillers showed higher FS and FM values in bis-GMA/TEGDMA resin composites. Twenty wt% BAG fillers showed no difference in FS and FM in UDMA/TEGDMA resin system. The addition of more than 20 wt% BAG fillers caused a marked decrease in FS and FM in both resin systems.
Marovic et al. [32]	The 10 wt% Cu-MBGN-loaded resin composites showed lower FS and higher FM and MH than the BAG-containing composites and the BAG-free control group.
Elamis et al. [33]	The silanization of BAG resulted in improved FS and FM while surface microhardness values decreased. The FS, FM, and MH of resin composites treated with alumina-doped 45S5 BAG were higher than those of the 45S5 BAG group.
Dieckmann et al. [34]	Microhardness was increased in 20 wt% BAG-loaded Filtek Bulk Fill, while no changes were found in Venus Bulk Fill or SDR composite materials.
Par et al. [35]	More than 10 wt% of BAG amount reduced the FS and MR, while 5 wt% BAG decreased FM. Higher amounts of BAG yielded a higher degradation of FS, FM, and MR after artificial aging in water.
Kattan et al. [38]	A decrease of 30% in the biaxial strength was observed for Ag-containing BAG-loaded composites.
Choi et al. [44]	Mesoporous BAG enhanced the microhardness and showed a slight decrease in the bond strength.
Kim et al. [45]	Ag- or Zn-doped BAG enhanced the MH and showed no significant difference in shear bond strength.
Korkut et al. [46]	The addition of S53P4 BAG fillers of less than 30 wt% did not affect FS and CS.
Chatziatavrou et al. [47]	Resin adhesive modified with Ag-doped BAG showed no significant difference in shear bond strength.
Lee et al. [48]	Zinc-doped phosphate-based glass (Zn-PBG) negatively affect the FS.
Lee et al. [49]	Graphene oxide-doped bioactive glass (GO-BAG) enhanced the microhardness and decreased shear bond strength decrease.
Khvostenko et al. [50]	FS, fracture toughness, and fatigue crack growth of 0–15 wt% Na-free BAG-loaded resin composites were superior to commercially available composite Heliomolar.
Hanif et al. [51]	The 45S5 BAG particle doped with 5, 10, and 15 wt% nanosilver resulted in reduced mechanical characteristics.
Han et al. [52]	Eight, 16, and 23 wt% BAG showed no adverse effects on FS and CS.
Proenca et al. [53]	BAG reflected the negative impact on MH.
Al-Eesa et al. [54]	The silanized BAG-loaded composites exhibited significantly higher CS and FS than the non-silanized BAG composites.

**Table 4:** The research conducted on the mineralization ability of BAG-loaded dental resin composites.

Research	Main Findings
Hu et al. [14]	The dentine surface was blocked by mineral components together with an increase in the microhardness of the dentin after 2 weeks of storage with resin composites incorporated with 65S BAG (65% Si, 31% Ca, and 4% P in mol%) in SBF and PBS.
Tezergil-Mutluay et al. [15]	F-containing phosphate-rich BAG-doped resin composites demonstrated a stronger ability to remineralize the demineralized dentin after storage in artificial saliva for 30 days compared to the BAG-loaded group.
Odermatt et al. [25]	Nano-BAG composite caused a final value of around 10.5 and produced numerous dispersed apatite. Micro-BAG composite raised the pH to a final value of 8.5 and created small and uniformly distributed crystals.
Elalmis et al. [33]	The Al-doped 45S5 BAG-containing resin composite showed decreased apatite formation after 28 days of immersion in SBF compared with conventional 45S5 BAG.
Par et al. [36]	CaF_2_-containing BAG composites showed final pH values in the range of 2.9–9.6 compared to 9.2–10.1 for LAS.
Balbinot et al. [37]	Niobium-doped BAG-loaded adhesive resins produced higher mineral deposition than the control group and BAG-containing composites after 28 days in SBF.
Choi et al. [44], Kim et al. [45], Lee et al. [49]	BAG fillers enhanced the remineralization and anti-demineralization length of enamel after submerging samples in demineralization solution for 6 h, followed by 18 h in remineralization solution for 14 days.
Chatzistavrou et al. [47]	Ag-doped BAG composites showed apatite-like phase on resin composites after 14 days of storage in SBF.
Lee et al. [48]	Resin composite containing Zn-doped phosphate-based bioactive glass increasingly released more Ca, P, Zn, and Na ions with a rise in Zn-BAG concentration.
Han et al. [52]	45S5 BAG-loaded resin composites demonstrated more mineralized layer occluding dentin tubules after immersion in SBF for 21 days.
Proenca et al. [53]	Resins containing 45S5 BAG had a high ability to alkalize the medium to ultimate pH values of around 10, to release calcium and phosphate ions and to produce a considerable volume of precipitates.
Al-Eesa et al. [54]	The silanized and non-silanized BAG modified composites had identical fluoride concentrations in artificial saliva until the 28-day time point. The non-silanized composite released higher fluoride from 28 days onwards. Silanization had no effect on the production of apatite
Par et al. [56]	The 20 wt% of BAG-loaded resin composites maintained the initial enamel microhardness for up to 5 acid addition cycles (20 d) and could maintain a plateau at pH = 9–10. The 20 wt% F-containing BAG and 10 wt% BAG kept the original enamel microhardness for 3 acid cycles (12 d) and showed only transient alkalization. The 10 wt% F-containing BAG group could maintain original enamel microhardness for 2 acid addition cycles (8 d).
Al-Eesa et al. [57]	The majority of the BAG particles in resin composites had reacted to create apatite in acidic artificial saliva at a pH of 4 (AS4). Faster degradation of the BAG disk was found in acidic media AS4. The formed fluorapatite signal was strongest for disks in the AS4 medium.
Kulkova et al. [58]	Bacterial biofilm growing medium led to apatite formation on the surfaces of the BAG composites.
Aponso et al. [59]	None of the BAG-containing composites showed more than a 0.2-unit pH shift using a scanning electrochemical microscopy (SECM) analytic approach.
Chatzistavrou et al. [60]	Apatite-like phase was detected on resin composites containing Ag-doped BAG after 14 days of storage in SBF.
Par et al. [61]	Dentine microhardness was maintained over different numbers of acid additions, as follows: 20 wt% BAG (up to 12 days), and 10 wt% BAG, and 20 wt% F-containing BAG (up to 4 days). The 20 wt% BAG group plateaued at pH = 9, other groups attained a stable pH at only 6–7.
Al-Eesa et al. [62]	The apatite crystals produced on the disk surface in artificial saliva at a pH of 7 were strongly oriented and grew in orientation over time.
Al-Eesa et al. [63]	The largest increase in pH (up to 3 pH levels) was found in the artificial saliva at a pH of 4 at the final time-point of 180 days.
Song et al. [64]	Gallium-doped mesoporous bioactive glass-modified resin composites revealed the increased release of Ca, P, and Ga ions along with a decrease in pH with immersion time
Brown et al. [65]	In the SBF at pH of 4, a final pH increase of 2 was seen. In the noncariogenic SBF at pH 7, a pH increase of 0.25 was observed.
Davis et al. [66]	Resin composite containing BAG fillers (BAG 81 (81% Si; 11% Ca; 4% P; 3% F;1% B)) released more fluoride ions after 222 h compared with BAG 61 (61% Si; 31% Ca; 4% P; 3% F; 1% B)). BAG 61 composites revealed more release of calcium ions during each of the 2- and 22-h time periods.
Fuss et al. [67]	The 10 wt% nano-sized (30–50 nm) bismuth (Bi)-modified 45S5 BAG-doped resin composites showed a final pH of 9.2 in hydrochloric acid (HAS).

**Table 5:** The research conducted on *in vitro* biological responses of BAG-loaded dental resin composites.

Research	Main Findings
Costa Lima Assad et al. [29]	No difference in human osteoblast (MG63) viability was found between the control group without composites and the experimental groups containing BAG-loaded resin composites using the direct-contact cell model.
Balbinot et al. [37]	Experimental adhesives containing 2 wt% BAG or 2 wt% niobium-doped BAG (BAGNb) reached levels of 126.89% and 129.53% of cell viability, respectively, compared with the control resin adhesive.
Kattan et al. [38]	The addition of 5, 10, and 15 wt% Ag-doped 58S BAG (Ag-BAG) in resin composites demonstrated improved antibacterial potentials against *S. mutans* and *L. casei.*
Choi et al. [44]	The addition of 5 wt% mesoporous BAG nanoparticles showed a significant antibacterial effect on both *S. mutans* and *P. gingivalis.*
Kim et al. [45], Lee et al. [48]	The resin composites incorporated with Zn-PBG revealed the antibacterial activity against *S. mutans.*
Korkut et al. [46]	Resin composites containing 5, 10, and 30 wt% of microparticulate S53P4 BAG significantly decreased the viability of *E. coli*, *S. aureus*, and *S. mutans.*
Chatzistavrou et al. [47]	The number of dead bacteria in the *S. mutans* biofilm significantly increased with the increase in Ag-doped BAG concentration in the resin composites.
Lee et al. [49]	BAG and graphene oxide (GO)-combined BAG had no effects on the cell viability of human gingival fibroblasts (HGF-1) using the direct-contact cell model. Resin composites containing a mixture of 5 wt% GO combined with BAG had a considerably stronger antibacterial impact on *S. mutans.*
Han et al. [52]	The 8 wt% 45S5 BAG-loaded resin composites were not cytotoxic to mouse fibroblast L929, while 16 wt% and 23 wt% BAG groups revealed cytotoxicity. The cell viability was returned to normal after adjusting the elevated pH value of the extracts to neutral.
Proenca et al. [53]	The resins containing 5, 10, and 20 wt% 45S5 BAG demonstrated a significant decrease in *S. mutans* viability, regardless of BAG levels.
Aponso et al. [59]	BAG composites demonstrated a reduced height and volume in *S. mutans* biofilm growth.
Chatzistarou et al. [60]	The composite containing 15 wt% Ag-BAG significantly inhibited *S. mutans* and *E. coli* growth.
Song et al. [64]	A 5 wt% gallium-doped mesoporous bioactive glass (GaMBN)-containing resin adhesive revealed significantly higher viability of human dental pulp stem cells (hDPSCs) than other groups after 24 h of cell culture. GaMBN-loaded resin adhesive showed no effects on the viability of *S. mutans.*
Khostenko et al. [71]	BAG-loaded resin composites showed lower average depth of bacterial penetration (61%) into the marginal gap in comparison to the control group, which showed 100% penetration.
Salehi et al. [72]	Resin composites containing 0, 5, 10, and 15 wt% of BAG impaired cell viability using composites extract with a dilution level of 1:8 or higher and under direct exposure to newly cured composites. Experimental composites after preincubation in a cell culture medium for 7 days showed similar cell metabolic activity to the control.

## Data Availability

Not Applicable.
